# Toll-like Receptor 4 Pathway Polymorphisms Interact with Pollution to Influence Asthma Diagnosis and Severity

**DOI:** 10.1038/s41598-018-30865-0

**Published:** 2018-08-23

**Authors:** Shepherd H. Schurman, Mercedes A. Bravo, Cynthia L. Innes, W. Braxton Jackson, John A. McGrath, Marie Lynn Miranda, Stavros Garantziotis

**Affiliations:** 1 Clinical Research Branch, Division of Intramural Research, National Institute of Environmental Health Sciences, National Institutes of Health, Research Triangle Park, North Carolina, 27709 United States; 2 Children’s Environmental Health Initiative, Rice University, Houston, Texas 77005 United States; 3 Social and Scientific Systems, Durham, North Carolina 27703 United States; 4 Department of Statistics, Rice University, Houston, Texas 77005 United States

## Abstract

Asthma is a common chronic lung disease, the incidence and severity of which may be influenced by gene-environment interactions. Our objective was to examine associations between single nucleotide polymorphisms (SNPs) and combinations of SNPs in the toll-like receptor 4 (TLR4) pathway, residential distance to roadway as a proxy for traffic-related air pollution exposure, and asthma diagnosis and exacerbations. We obtained individual-level data on genotype, residential address, and asthma diagnosis and exacerbations from the Environmental Polymorphisms Registry. Subjects (n = 2,704) were divided into three groups (hyper-responders, hypo-responders, and neither) based on SNP combinations in genes along the TLR4 pathway. We geocoded subjects and calculated distance, classified as <250 m or ≥250 m, between residence and nearest major road. Relationships between genotype, distance to road, and odds of asthma diagnosis and exacerbations were examined using logistic regression. Odds of an asthma diagnosis among hyper-responders <250 m from a major road was 2.37(0.97, 6.01) compared to the reference group (*p* < 0.10). Hypo-responders ≥250 m from the nearest road had lower odds of activity limitations (0.46 [0.21, 0.95]) and sleeplessness (0.36 [0.12, 0.91]) compared to neither-responders (*p* < 0.05). Specific genotype combinations when combined with an individual’s proximity to roadways, possibly due to traffic-related air pollution exposure, may affect the likelihood of asthma diagnosis and exacerbations.

## Introduction

Asthma is a chronic disease characterized by airway inflammation and narrowing. In recent decades, asthma prevalence has increased^1^ and it has become a significant public health problem in the US, affecting quality of life, work absenteeism, health care utilization, and health expenditures^2,3^.

Gene-environment interactions play an important role in the development and severity of asthma^4^. Air pollutants activate innate immunity, particularly the TLR4 pathway^5,6^. This is especially true with ozone and nitrogen dioxide but has been shown with other air pollutants such as vanadate^7^. TLR4 recognizes lipopolysaccharide (LPS) and protects against gram-negative infection. There is evidence that traffic pollutants can interfere with LPS-induced signaling^8^. Maladaptive activation of TLR4 can importantly contribute to acute or chronic inflammation^9^. SNPs that perturb TLR4 responses are associated with asthma^10–14^.

Precision medicine seeks to personalize predictive and therapeutic interventions by accounting for individual genetic, exposure, and disease profiles. However, little attention has been paid to personalized gene-environment interactions in complex diseases like asthma. We, and others^15^, hypothesized that population-level data may be exploited to generate predictions for individual gene-environment interactions. To test a proof-of-principle of this hypothesis with asthma, we examined associations between combinations of specific SNPs along the TLR4 inflammatory pathway, air pollution exposure, and asthma-related health outcomes. We reasoned that individuals who carry SNP combinations rendering them hyper-responsive to TLR4 activation may be particularly sensitive to air pollution exposures. Conversely, individuals with SNP combinations that render them hypo-responsive to TLR4 activation may be less sensitive to air pollution exposures.

We examined three genes in the TLR4 complex (*TLR4*, *CD14* and *TIRAP*) and *TNFα* which is activated downstream of TLR4. SNPs were selected based on relatively high minor allele frequency (MAF) and previous evidence relating the minor alleles to differential health outcomes. They included two putative gain-of-function SNPs (rs2569190 in *CD14* and rs1800629 in *TNFα*) and two putative loss-of-function SNPs (rs4986791 in *TLR4* and rs8177374 in *TIRAP*). The *CD14* SNP minor allele (MAF up to 22%) is associated with higher circulating CD14 levels and asthmatic exacerbations^10^. The *TNFα* SNP minor allele, rs1800629 (MAF up to 15%), leads to increased *TNFα* transcriptional activation^16^, and is associated with chronic obstructive pulmonary disease^17^. A synergistic effect has been shown between polymorphisms of *TNFα* and *CD14* and bronchial responsiveness in children with asthma^18^. The *TLR4* SNP minor allele rs4986790, which is in linkage disequilibrium with rs4986791 (MAF up to 6.6% in Caucasians), confers hypo-responsiveness to inhaled LPS^19^. The *TIRAP* SNP minor allele rs8177374 is associated with decreased response to LPS^20^. A synergistic effect has been shown between polymorphisms of *TLR4* and *TIRAP* in risk for severe infections following cardiac surgery^21^.

We explored possible interactions between air pollution exposure and underlying genotype by examining the residential distance to nearest road as a proxy for traffic-related air pollution exposure. Our outcomes were likelihood of asthma diagnosis and asthma exacerbations. We hypothesized that: (1) asthma exacerbations are associated with specific genotype combinations; and (2) roadway proximity, as a proxy for air pollution exposure, may act as an effect modifier in the relationship between genotype and asthma.

## Results

### Individual SNP genotype associations with asthma diagnosis

We first examined the relationship of genotype and asthma at the individual SNP/gene level (*TLR4*, *CD14, TIRAP*, and *TNFα*). All SNPs followed Hardy-Weinberg Equilibrium for the control (no asthma) samples.

Tables 1 and 2 show the number and percentage of individuals with each SNP by sex, race, ethnicity, smoking status, and income. Table 3 shows mean age of asthma diagnosis by SNP. There were no significant differences in mean age of asthma diagnosis for *TNFα* and *CD14* or for *TLR4* heterozygotes. There were too few individuals homozygous for the *TLR4* minor allele to perform this comparison. The mean age of asthma diagnosis was significantly younger for individuals with the wildtype (WT) *TIRAP* allele (22.4 years) compared to those who are carriers for this allele (26.1 years; *p* = 0.008) indicating that the onset of asthma may be delayed in individuals with at least one copy of the minor *TIRAP* allele of rs8177374.

**Table 1:** Cohort statistics for phenotypic characteristics by *TNFα* and *CD14* SNPs based on asthma diagnosis.

	No Asthma (1741)^a^			Asthma (963)		
	WT	Het	Minor	WT	Het	Minor
	N (%)	N (%)	N (%)	N (%)	N (%)	N (%)
***TNFα*** **rs1800629**						
*Sex* ^b^						
Male	538 (69.9)	209 (27.1)	23 (3.0)	169 (68.4)	73 (29.6)	5 (2.0)
Female	684 (70.4)	268 (27.6)	19 (2.0)	515 (71.9)	187 (26.1)	14 (2.0)
*Race*						
White	748 (69.0)	303 (28.0)	33 (3.0)	451 (69.3)	185 (28.4)	15 (2.3)
BAA	418 (71.8)	156 (26.8)	8 (1.4)	176 (72.1)	64 (26.2)	4 (1.6)
Other	56 (74.7)	18 (24.0)	1 (1.3)	57 (83.8)	11 (16.2)	0 (0.0)
*Ethnicity*						
Non-Hispanic	1105 (69.5)	446 (28.1)	39 (2.5)	641 (71.1)	244 (27.1)	17 (1.9)
Hispanic	91 (79.1)	22 (19.1)	2 (1.7)	24 (70.6)	9 (26.5)	1 (2.9)
Unknown	26 (72.2)	9 (25.0)	1 (2.8)	19 (70.4)	7 (25.9)	1 (3.7)
*Smoker*						
Never	690 (71.0)	261 (26.9)	21 (2.2)	382 (70.3)	152 (28.0)	9 (1.7)
Ever	532 (69.2)	216 (28.1)	21 (2.7)	302 (71.9)	108 (25.7)	10 (2.4)
*Income ($/year)*						
≥$60,000	515 (70.0)	199 (27.0)	22 (3.0)	274 (70.8)	105 (27.1)	8 (2.1)
$40,000–$59,999	250 (73.3)	83 (24.3)	8 (2.3)	122 (71.8)	44 (25.9)	4 (2.4)
$20,000–$39,999	277 (70.5)	109 (27.7)	7 (1.8)	140 (71.8)	51 (26.2)	4 (2.1)
<$20,000	180 (66.4)	86 (31.7)	5 (1.8)	148 (70.1)	60 (28.4)	3 (1.4)
*BMI Category (kg/m*^2^)						
under 18.5	14 (82.4)	3 (17.6)	0 (0.0)	8 (66.7)	4 (33.3)	0 (0.0)
18.5–24.9	324 (72.3)	110 (24.6)	14 (3.1)	171 (68.4)	73 (29.2)	6 (2.4)
25–29.9	416 (69.3)	172 (28.7)	12 (2.0)	186 (73.5)	63 (24.9)	4 (1.6)
≥30	454 (69.2)	187 (28.5)	15 (2.3)	308 (71.3)	115 (26.6)	9 (2.1)
***CD14*** **rs2569190**						
*Sex*						
Male	232 (30.1)	380 (49.4)	158 (20.5)	72 (29.1)	125 (50.6)	50 (20.2)
Female	334 (34.4)	440 (45.3)	197 (20.3)	204 (28.5)	367 (51.3)	145 (20.3)
*Race*						
White	286 (26.4)	537 (49.5)	261 (24.1)	149 (22.9)	356 (54.7)	146 (22.4)
BAA	259 (44.5)	254 (43.6)	69 (11.9)	107 (43.9)	103 (42.2)	34 (13.9)
Other	21 (28.0)	29 (38.7)	25 (33.3)	20 (29.4)	33 (48.5)	15 (22.1)
*Ethnicity*						
Non-Hispanic	530 (33.3)	749 (47.1)	311 (19.6)	262 (29.0)	458 (50.8)	182 (20.2)
Hispanic	29 (25.2)	51 (44.3)	35 (30.4)	6 (17.6)	21 (61.8)	7 (20.6)
Unknown	7 (19.4)	20 (55.6)	9 (25.0)	8 (29.6)	13 (48.1)	6 (22.2)
*Smoker*						
Never	327 (33.6)	453 (46.6)	192 (19.8)	156 (28.7)	289 (53.2)	98 (18.0)
Ever	239 (31.1)	367 (47.7)	163 (21.2)	120 (28.6)	203 (48.3)	97 (23.1)
*Income ($/year)*						
≥$60,000	242 (32.9)	338 (45.9)	156 (21.2)	85 (22.0)	219 (56.6)	83 (21.4)
$40,000–$59,999	97 (28.4)	162 (47.5)	82 (24.0)	61 (35.9)	75 (44.1)	34 (20.0)
$20,000–$39,999	136 (34.6)	190 (48.3)	67 (17.0)	60 (30.8)	93 (47.7)	42 (21.5)
<$20,000	91 (33.6)	130 (48.0)	50 (18.5)	70 (33.2)	105 (49.8)	36 (17.1)
*BMI Category (kg/m*^2^)						
under 18.5	4 (23.5)	8 (47.1)	5 (29.4)	2 (16.7)	9 (75.0)	1 (8.3)
18.5–24.9	141 (31.5)	208 (46.4)	99 (22.1)	60 (24.0)	130 (52.0)	60 (24.0)
25–29.9	189 (31.5)	289 (48.2)	122 (20.3)	73 (28.9)	134 (53.0)	46 (18.2)
≥30	225 (34.3)	306 (46.6)	125 (19.1)	136 (31.5)	210 (48.6)	86 (19.9)

^a^The total number of individuals is 2,704. Of those, 1,688 reported no asthma and 53 did not respond, for a total of 1,741 “No asthma”, and 963 reported having a diagnosis of asthma.

^b^Percentages presented in the following rows are percentages based on the genotype total for either no asthma or asthma. Totals may differ based on non-response to the specific characteristic.

**Table 2:** Cohort statistics for phenotypic characteristics by *TLR4* and *TIRAP* SNPs based on asthma diagnosis.

	No Asthma (1,741)^a^			Asthma (963)		
	WT	Het	Minor	WT	Het	Minor
	N (%)	N (%)	N (%)	N (%)	N (%)	N (%)
***TLR4*** **rs4986791**						
*Sex*						
Male	704 (91.4)	66 (8.6)	0 (0.0)	219 (88.7)	28 (11.3)	0 (0.0)
Female	894 (92.1)	75 (7.7)	2 (0.2)	652 (91.1)	63 (8.8)	1 (0.1)
*Race*						
White	964 (88.9)	119 (11)	1 (0.1)	571 (87.7)	79 (12.1)	1 (0.2)
Black/African-American	564 (96.9)	18 (3.1)	0 (0.0)	237 (97.1)	7 (2.9)	0 (0.0)
Other	70 (93.3)	4 (5.3)	1 (1.3)	63 (92.6)	5 (7.4)	0 (0.0)
*Ethnicity*						
Non-Hispanic	1458 (91.7)	131 (8.2)	1 (0.1)	813 (90.1)	88 (9.8)	1 (0.1)
Hispanic	106 (92.2)	8 (7.0)	1 (0.9)	33 (97.1)	1 (2.9)	0 (0.0)
Unknown	34 (94.4)	2 (5.6)	0 (0.0)	25 (92.6)	2 (7.4)	0 (0.0)
*Smoker*						
Never	897 (92.3)	75 (7.7)	0 (0.0)	492 (90.6)	50 (9.2)	1 (0.2)
Ever	701 (91.2)	66 (8.6)	2 (0.3)	379 (90.2)	41 (9.8)	0 (0.0)
*Income ($/year)*						
≥$60,000	667 (90.6)	69 (9.4)	0 (0.0)	344 (88.9)	43 (11.1)	0 (0.0)
$40,000–$59,999	309 (90.6)	31 (9.1)	1 (0.3)	154 (90.6)	16 (9.4)	0 (0.0)
$20,000–$39,999	364 (92.6)	29 (7.4)	0 (0.0)	173 (88.7)	21 (10.8)	1 (0.5)
<$20,000	258 (95.2)	12 (4.4)	1 (0.4)	200 (94.8)	11 (5.2)	0 (0.0)
*BMI Category (kg/m*^2^)						
under 18.5	17 (100.0)	0 (0.0)	0 (0.0)	10 (83.3)	2 (16.7)	0 (0.0)
18.5–24.9	407 (90.8)	40 (8.9)	1 (0.2)	230 (92.0)	20 (8.0)	0 (0.0)
25–29.9	546 (91.0)	53 (8.8)	1 (0.2)	223 (88.1)	30 (11.9)	0 (0.0)
≥30	610 (93.0)	46 (7.0)	0 (0.0)	393 (91.0)	38 (8.8)	1 (0.2)
***TIRAP*** **rs8177374**						
*Sex*						
Male	589 (76.5)	166 (21.6)	15 (1.9)	191 (77.3)	54 (21.9)	2 (0.8)
Female	792 (81.6)	173 (17.8)	6 (0.6)	543 (75.8)	161 (22.5)	12 (1.7)
*Race*						
White	780 (72.0)	283 (26.1)	21 (1.9)	460 (70.7)	177 (27.2)	14 (2.2)
Black/African-American	543 (93.3)	39 (6.7)	0 (0.0)	226 (92.6)	18 (7.4)	0 (0.0)
Other	58 (77.3)	17 (22.7)	0 (0.0)	48 (70.6)	20 (29.4)	0 (0.0)
*Ethnicity*						
Non-Hispanic	1262 (79.4)	308 (19.4)	20 (1.3)	686 (76.1)	202 (22.4)	14 (1.6)
Hispanic	89 (77.4)	26 (22.6)	0 (0.0)	28 (82.4)	6 (17.6)	0 (0.0)
Unknown	30 (83.3)	5 (13.9)	1 (2.8)	20 (74.1)	7 (25.9)	0 (0.0)
*Smoker*						
Never	787 (81.0)	177 (18.2)	8 (0.8)	410 (75.5)	126 (23.2)	7 (1.3)
Ever	594 (77.2)	162 (21.1)	13 (1.7)	324 (77.1)	89 (21.2)	7 (1.7)
*Income ($/year)*						
≥$60,000	565 (76.8)	161 (21.9)	10 (1.4)	282 (72.9)	98 (25.3)	7 (1.8)
$40,000–$59,999	278 (81.5)	60 (17.6)	3 (0.9)	126 (74.1)	42 (24.7)	2 (1.2)
$20,000–$39,999	314 (79.9)	73 (18.6)	6 (1.5)	153 (78.5)	39 (20.0)	3 (1.5)
<$20,000	224 (82.7)	45 (16.6)	2 (0.7)	173 (82.0)	36 (17.1)	2 (0.9)
*BMI Category (kg/m*^2^)						
under 18.5	12 (70.6)	5 (29.4)	0 (0.0)	9 (75.0)	2 (16.7)	1 (8.3)
18.5–24.9	342 (76.3)	99 (22.1)	7 (1.6)	185 (74.0)	61 (24.4)	4 (1.6)
25–29.9	488 (81.3)	107 (17.8)	5 (0.8)	188 (74.3)	63 (24.9)	2 (0.8)
≥30	520 (79.3)	127 (19.4)	9 (1.4)	339 (78.5)	86 (19.9)	7 (1.6)

^a^The total number of individuals is 2,704. Of those, 1,688 reported no asthma and 53 did not respond, for a total of 1,741 “No asthma”, and 963 reported having a diagnosis of asthma.

^b^Percentages presented in this row and following rows are percentages based on the genotype total for either no asthma or asthma.

**Table 3:** Mean Age of Asthma Diagnosis of Geocoded Participants with Asthma.

Genotype	N*	Mean (years)	SD	t-test	*P*-value
***TNFα*** **rs1800629**					
GG	658	23.4	18.4		
GA + AA	265	22.8	18.5	−0.42	0.67
***CD14*** **rs2569190**					
CC	263	24.1	18.3		
CT + TT	660	22.9	18.4	−0.86	0.39
***TLR4*** **rs4986791**					
CC	834	23.0	18.2		
CT + TT	89	25.5	19.9	1.21	0.23
*TIRAP* **rs8177374**					
CC	703	22.4	18.0		
CT + TT	220	26.1	19.6	2.66	**0.008**

*Total number of participants is 923 for each SNP.

Results are from t-test comparisons of the mean of the SNP carrier compared to its appropriate WT.

We did not detect significant differences in the proportions of carriers vs. WT for each SNP between individuals with and without asthma, using χ^2^ tests. Allele frequencies were also examined and showed no significant differences.

### Individual SNP genotype associations with asthma exacerbations

There were no significant differences in reports of asthma-related sleeplessness or activity limitations by SNP during the previous 14-days (Tables S1–S4). *TLR4* carriers vs. WT had lower odds ratio (OR) of asthma-related emergency room (ER) visits in the past 12-months (0.39 [0.15, 0.97]) in an unadjusted model. This association was not significant after adjustment for sex, race, smoking status, body mass index (BMI) and income.

### Association of responder status, asthma diagnosis and exacerbations, and distance to road

Of the 2,704 participants classified by responder status, 369 (13.7%) were hyper-responders [carriers of *CD14* and *TNFa* minor SNPs] and [WT for *TLR4* and *TIRAP* SNPs], 132 (4.9%) hypo-responders [carriers of *TLR4* and/or *TIRAP* minor SNPs] and [WT for *CD14* and *TNFa* SNPs], and 2,203 (81.5%) neither [all others]. There was no significant variation in the distribution of the responder categories among those with and without asthma (Table S5**)**, no difference in age of asthma diagnosis (Table S6**)**, or in prevalence of asthma-related exacerbations in 14-days (Table S7**)** or 12-months (Table S8**)**.

**Figure 1:**
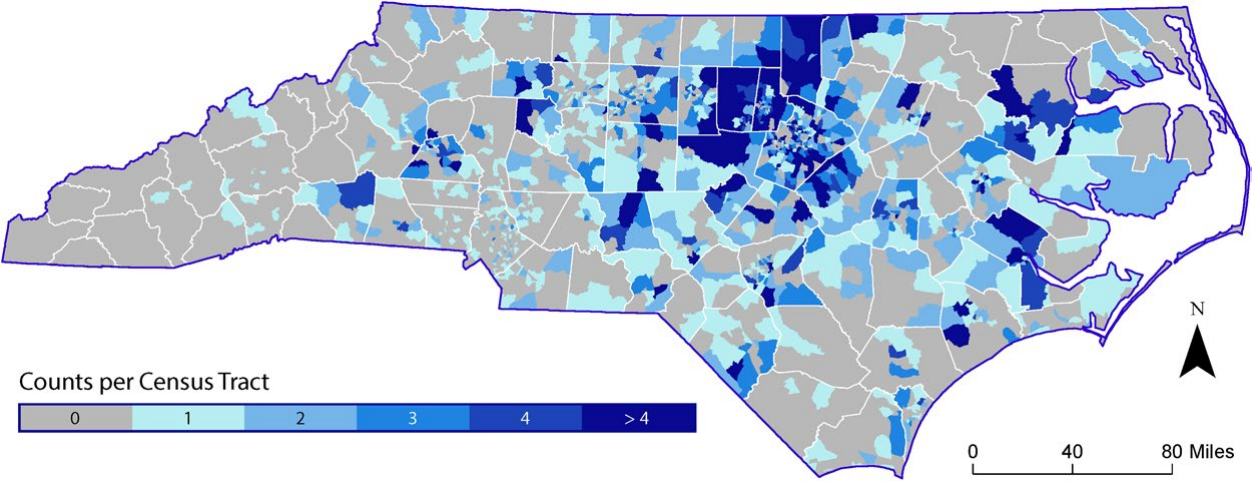
Genotyped EPR Cohort Distribution based on Residential Address, North Carolina.

Summary information for the individuals in our analysis, by asthma diagnosis and exacerbation, is provided in Table 4. For distance to road analyses, 36 participants were not used because of missing BMI data (2,668 participants analyzed). Figure 1 shows the distribution of participants at the county level across census tracts within North Carolina, the state with the majority (94%) of participants. Mean (median) distance to primary and secondary roads was 6.91 km (3.67 km) and 3.98 km (2.90 km), respectively. Classifying proximity in this way, 200 (7.5%) subjects lived <250 m from the nearest road and 2,468 (92.5%) subjects lived ≥250 m from the nearest road. Distributions of covariates (sex, race, smoking status, BMI, income) by responder-type and distance to roadway categorization are provided in Tables S9 and S10. African-Americans were less likely to be hypo-responders (2.4%) compared to Caucasians (5.5%), and a higher percentage of African-Americans (8.9%) resided in the <250 m category compared to Caucasians (6.9%, *p* = 0.10). Percentages of hyper-, hypo-, and neither-responders residing in the <250 m distance category were not significantly different.

**Table 4:** Summary statistics of the study population.

All observations (n = 2,668)^a^	Asthma diagnosis^b^		Asthma exacerbations^c^			
	No N (%)	Yes N (%)	Activity limitations N (%)	Sleeplessness N (%)	Emergency room visits N (%)	Any N (%)
	1,721 (64.5)	947 (35.5)	325 (34.3)	208 (21.9)	115 (12.1)	388 (41.0)
**Sex** ^d^						
Female	958 (55.7)	705 (74.4)	261 (80.3)	180 (86.5)	92 (80.0)	314 (81.0)
Male	763 (44.3)	242 (25.6)	64 (19.7)	28 (13.5)	23 (20.0)	74 (19.0)
**Race**						
Black/African-American	572 (33.2)	235 (24.8)	88 (27.1)	81 (38.9)	50 (43.5)	245 (63.1)
Caucasian	1,073 (62.3)	644 (68.0)	212 (65.2)	114 (54.8)	59 (51.3)	112 (28.9)
Other	76 (4.42)	68 (7.18)	25 (7.69)	13 (6.25)	6 (5.22)	31 (8.00)
**Ethnicity**						
Hispanic	113 (6.57)	34 (3.59)	11 (3.38)	6 (2.88)	2 (1.74)	13 (3.35)
Non-Hispanic	1,575 (91.5)	887 (93.7)	304 (93.5)	195 (93.8)	110 (95.6)	364 (93.8)
Unknown/not reported	33 (1.92)	26 (2.74)	10 (3.08)	7 (3.37)	3 (2.61)	11 (2.84)
**Distance to nearest road (primary or secondary)**						
<250 m	121 (7.03)	79 (8.34)	23 (7.08)	17 (8.17)	7 (6.09)	27 (6.96)
≥250 m	1,600 (92.9)	868 (91.7)	302 (92.9)	191 (91.8)	108 (93.9)	361 (93.0)
**Distance to nearest road (primary, secondary, or tertiary)**						
<250 m	722 (42.0)	436 (46.0)	152 (46.8)	104 (50.0)	57 (49.6)	180 (46.4)
≥250 m	999 (58.0)	511 (54.0)	173 (523.2)	104 (50.0)	58 (50.4)	208 (53.6)
**Responder type (genetic profile)**						
Hyper	228 (13.2)	135(14.3)	52 (16.0)	28 (13.5)	15 (13.0)	60 (15.5)
Hypo	87 (5.06)	43 (4.54)	10 (3.08)	5 (2.40)	3 (2.61)	13 (3.35)
Neither	1,406 (81.7)	769 (81.2)	263 (80.9)	175 (84.1)	97 (84.3)	315 (81.2)
**Income ($/year)**						
<$20,000	264 (15.3)	204 (21.5)	106 (32.6)	86 (41.3)	56 (48.7)	126 (32.5)
$20,000–$39,999	389 (22.6)	192 (20.3)	83 (25.5)	54 (26.0)	26 (22.6)	92 (23.7)
$40,000–$59,000	338 (19.6)	164 (17.3)	46 (14.2)	24 (11.5)	11 (9.56)	55 (14.2)
≥$60,000	730 (42.4)	387 (40.9)	90 (27.7)	44 (21.2)	22 (19.1)	115 (29.6)
**Body Mass Index (BMI)**						
Overweight or obese	1,256 (73.0)	685 (72.3)	249 (76.6)	169 (81.3)	94 (81.7)	300 (77.3)
**Smoking status**						
Ever	756 (43.9)	415 (43.8)	163 (50.2)	90 (43.3)	51 (44.3)	199 (51.3)
Never	965 (56.1)	532 (56.2)	162 (49.8)	118 (56.7)	64 (55.7)	189 (48.7)
**Average age at asthma diagnosis (years)** ^e^	—	22.4	26.2	25.2	23.5	25.4

^a^The total number of geocoded individuals in the initial dataset was n = 2,830. Individuals with missing responder type (n = 16) or missing covariates (n = 146) were excluded from the dataset (final n = 2,668).

^b^Yes/no categories of asthma diagnosis are mutually exclusive.

^c^The asthma exacerbation categories are not mutually exclusive: an individual may have reported more than one asthma exacerbation. Only individuals with an asthma diagnosis (n = 947) reported asthma exacerbations. Any asthma exacerbation indicates that an individual reported one or more of the three specific asthma exacerbations.

^d^Percentages presented in this row and following rows are percentages based on the column total (i.e., denominator = 1,721 for percentages in the “No asthma diagnosis” column and denominator = 947 for percentages in the “Asthma diagnosis” column).

^e^This row only applies to individuals with an asthma diagnosis.

We examined asthma diagnosis, with distance to roadway and responder-type as the exposures of interest, using a logistic regression model. Unadjusted and adjusted ORs for the association of asthma diagnosis with responder-type and distance-to-roadway are presented in Table 5. Adjusted models controlled for smoking status, sex, race, BMI, and income. The likelihood ratio test comparing interaction and adjusted models for asthma diagnosis provided the suggestion of an interaction between responder-type and distance to road (*p* = 0.12). Thus, we focus on results from interaction models for asthma diagnosis. The reference group was chosen as a sub-group representative of the source population: Caucasian, female, non-smoking, high income (≥$60,000/year), BMI in the normal or underweight range (i.e. not obese or overweight), neither-responders, and residing ≥250 m from the nearest major (primary or secondary) road.

**Table 5:** Asthma diagnosis, responder status, and distance to road.

	Unadjusted^b^ OR (95% CI)	Adjusted^c^ OR (95% CI)	Interaction^d^ OR (95% CI)
<250 m^a^	*1.22 (0.91, 1.64)* ^+^	1.18 (0.87, 1.59)	
Hyper-responder	1.07 (0.85, 1.35)	1.08 (0.85, 1.36)	
Hypo-responder	0.91 (0.62, 1.31)	0.84 (0.57, 1.23)	
Ever smoker	—	0.99 (0.83, 1.17)	
Male	—	0.41 (0.34, 0.49)*	
Black/African American	—	0.57 (0.47, 0.69)*	
Other races	—	*1.40 (0.98, 1.99)* ^+^	
Income < $20,000	—	1.58 (1.24, 2.01)*	
Income $20,000-$39,999	—	0.94 (0.75, 1.18)	
Income $40,000-$59,999	—	0.95 (0.75, 1.19)	
Overweight or obese, based on body mass index (BMI)	—	1.11 (0.92, 1.34)	
<250 m distance(Hyper)	—	—	*2.37 (0.97, 6.01)* ^+^
<250 m distance(Hypo)	—	—	1.61 (0.39, 6.48)

^a^Distance to roadway is classified as <250 m and ≥250 m from nearest primary or secondary road.

^b^Unadjusted models included only responder and distance categories.

^c^Adjusted models included responder, distance category, sex, race, smoking status, body mass index, and income categories.

^d^Interaction models included all covariates in adjusted models in addition to an interaction term for distance and responder type.

^*^Indicates significance at *p* < 0.05.

^+^Indicates *p* < 0.10.

Both hyper- and hypo-responders ≥250 m from a major road had similar odds of reporting an asthma diagnosis as neither-responders ≥250 m from a major road (reference group). The OR of an asthma diagnosis among hyper-responders <250 m from a major road was 2.37 (0.97, 6.01) compared to the reference group. Holding other variables constant at their reference value, the OR of asthma diagnosis among individuals with incomes <$20,000 was 1.58 (1.24, 2.01) compared to individuals with incomes ≥$60,000. African-Americans and men were less likely to report an asthma diagnosis, with adjusted ORs of 0.57 (0.47, 0.69) and 0.41 (0.34, 0.49), respectively. The OR of an asthma diagnosis among other races was 1.40 (0.98, 1.99) compared to Caucasians.

### Asthma exacerbations

For individuals that reported an asthma diagnosis, we examined road proximity, responder-type, and each asthma exacerbation (asthma-related activity limitations, sleeplessness, and ER visits) using logistic regression. Additionally, we looked at the outcome of “any” asthma exacerbation, i.e., individuals reporting any one or more of these three exacerbations. We fit unadjusted, adjusted, and interaction logistic regression models for each asthma exacerbation. The likelihood ratio test indicated that there was no evidence of interaction between responder-type and distance to road on any of the asthma exacerbations examined; here we present results from the adjusted model (Table 6).

**Table 6:** Asthma exacerbations, responder status, and distance to road^a,b^.

	Activity limitations OR (95% CI)	Sleeplessness OR (95% CI)	Emergency room visits OR (95% CI)	Any exacerbations OR (95% CI)
<250 m distance	0.69 (0.40, 1.15)	0.80 (0.42, 1.44)	0.53 (0.21, 1.16)	0.63 (0.37, 1.07)
Hyper-responder	1.29 (0.86, 1.93)	0.92 (0.55, 1.48)	0.86 (0.45, 1.54)	1.23 (0.83, 1.83)
Hypo-responder	0.46 (0.21, 0.95)*	0.36 (0.12, 0.91)*	0.47 (0.11, 1.42)	0.50 (0.24, 0.99)*
Smoker	*1.26 (0.95, 1.68)* ^+^	1.72 (1.23, 2.42)*	1.35 (0.88, 2.05)	*1.22 (0.94, 1.61)* ^+^
Male	0.63 (0.45, 0.88)*	0.38 (0.24, 0.59)*	0.70 (0.42, 1.14)	0.56 (0.40, 0.77)*
African American	0.77 (0.54, 1.09)	1.57 (1.07, 2.29)*	1.71 (1.08, 2.68)*	0.96 (0.68, 1.35)
Other races	1.17 (0.67, 2.03)	1.12 (0.54, 2.16)	0.90 (0.33, 2.11)	1.38 (0.80, 2.37)
Income < $20,000	3.79 (2.56, 5.64)*	4.56 (2.91, 7.24)*	5.01 (2.91, 9.13)*	3.84 (2.61, 5.69)*
Income $20,000-$39,999	2.61 (1.77, 3.88)*	2.36 (1.48, 3.79)*	2.07 (1.11, 3.89)*	2.10 (1.44, 3.07)*
Income $40,000-$59,999	1.36 (0.89, 2.07)	1.24 (0.71, 2.13)	1.09 (0.49, 2.27)	1.22 (0.81, 1.82)
Overweight or obese, based on body mass index (BMI)	1.44 (1.05, 2.01)*	1.75 (1.18, 2.67)*	1.68 (1.02, 2.90)*	1.58 (1.15, 2.17)*

^a^Distance to roadway considers nearest primary and secondary road.

^b^Results reported are for adjusted models, which include sex, race, smoking status, body mass index, and income category as covariates.

*Indicates significance at *p* < 0.05.

^+^Indicates *p* < 0.10.

Hyper-responders ≥250 m from a major road had similar odds of reporting any/all of the asthma exacerbations examined as neither-responders (Table 6). Hypo-responders ≥250 m from the nearest road, however, had lower odds of reporting activity limitations (0.46 [0.21, 0.95]), sleeplessness (0.36 [0.12, 0.91]), and “any” exacerbations (0.50 [0.24, 0.99]) compared to neither-responders ≥250 m from the nearest road (all *p* < 0.05).

Holding other variables constant at their reference value, the OR of sleeplessness among smokers was 1.72 (1.23, 2.42) compared to nonsmokers. Odds of activity limitations among smokers was suggestively higher (1.26 [0.95, 1.68]) when compared to nonsmokers. Men were less likely to report activity limitations, sleeplessness, and “any” exacerbation compared to women. African-Americans were more likely to report sleeplessness (1.57 [1.07, 2.29]) and ER visits (1.71 [1.08, 2.68]) than Caucasians. Odds of all of the exacerbations examined were higher among individuals with incomes <$20,000/year and individuals with incomes $20,000 to $39,999/year compared to individuals with income ≥$60,000/year. Also, odds of all of the exacerbations examined were higher among individuals who were overweight or obese, based on BMI, compared to individuals who were normal weight or underweight.

### Sensitivity analysis

We repeated our main analysis but classified proximity to road as: (1) <300 m or ≥300 m, <400 m or ≥400 m and <500 m and ≥500 m from a primary or secondary road; and (2) <250 m or ≥250 m from a primary, secondary, or tertiary road. Tertiary roads included local, neighborhood, and rural roads^22^. Overall, findings from the sensitivity analyses were generally consistent with those of the main analysis: hypo-responders had lower odds of sleeplessness, activity limitations, and “any” exacerbation compared to neither-responders (Table S11). Full results of the sensitivity analysis are presented in Tables S11–S17.

## Discussion

This study set out to test, as a proof-of-principle, the ability to use refined population analysis of gene-environment interactions to create personalized predictions for the activity of a complex disease.

We first explored relationships between genotype and asthma. There was some evidence that individual SNPs related to asthma diagnosis and exacerbations. For example, the mean age of asthma diagnosis was 4 years lower for individuals with the WT *TIRAP* allele compared to subjects carrying the minor allele. This indicates that rs8177374 is a loss-of-function allele that may delay, but not prevent, development of asthma, and is in itself a novel finding.

We then explored relationships between air pollution exposure and genotype combinations by examining asthma exacerbations, responder profiles, and residential distance to nearest road. We hypothesized that distance to roadway and responder status would interactively be associated with likelihood of asthma diagnosis and exacerbations. Consistent with this hypothesis, we found that hypo-responders had significantly lower odds of reporting activity limitations, sleeplessness, and “any” asthma exacerbation compared to neither-responders. Additionally, we found a suggestive interaction between genotype and distance-to-road on odds of asthma diagnosis for hyper-responders.

There has been limited research on human innate immune gene SNPs in relation to pollution exposure and airway inflammation. Most studies focus on children: there is a positive association between the *TNFα* SNP rs1800629 and allergic rhinitis^23,24^, a protective effect of the *TLR4* SNP rs4986791 in the development of hay fever^25^, and an interaction of the *TLR4* SNPs with exposure to particulate matter for prevalence of asthma^13^. In adults, the role of the TLR4 pathway in association with pollution exposure is much less defined^26^. Our findings extend the existing literature significantly because our study was conducted in adults and evaluated interaction of pollution with a signaling pathway, an approach that is thought to be more powerful^27^.

Precision medicine should take into account both genetic and exposure profiles. Genetic influences can be assessed with unbiased testing of random genetic and epistatic effects, as happens in large genome-wide association studies. We tested another approach, which analyzes pathways based on strong scientific support for relevance in disease pathogenesis. Such pathway-wide analyses may require smaller sample sizes to detect an effect. Furthermore, by taking into account the additive effect of genetic polymorphisms along the same pathway, the sensitivity of genetic analysis can be amplified. In addition, if we further selectively investigate environmental influences that are known to interact with the genetic pathways assessed, we may be able to create intelligent predictive models of gene-environment interactions that can be used on an individual basis. One can imagine an iterative process of analysis of several such pathways, which would result in an algorithmic approach to treatment of complex environmental disease.

Most published reports focus on rare gene variants with strong effects. This approach has great strengths but also limitations, especially in polygenic, complex diseases. For example, previously identified polymorphisms from several large genome-wide association studies only explain a fraction of the variability in lung function^28^ and are associated with a minority of asthma or chronic obstructive pulmonary disease cases^29^. Furthermore, it can be argued that the genetic background for many diseases comes from a combination of several, fairly common, weakly active SNPs, which can co-exist in the same individual and additively create a strong pull toward a particular effect^30^. Our pathway-focused approach served to test this hypothesis.

Taking into account environmental exposures, which are specifically linked to the pathway analyzed, enhances the approach. For example, Romieu and London^31,32^ studied common loss-of-function SNPs in the antioxidant genes *GSTM1* and *GSTP1*, and their effects on the lung function of asthmatic children exposed to high ozone levels. In a series of elegant papers, these investigators showed that asthmatic children with the *GSTM1* or *GSTP1* null allele were at especially high risk of lung function decrements following exposure to ozone, suggesting a gene-environment interaction. Patients who carried the null allele for both *GSTM1* and *GSTP1* had even higher risk of ozone-induced lung function effects, suggesting epistatic interactions along the anti-oxidant pathway.

Our study has several limitations. We used road classifications rather than traffic volume or measurements of air pollutant concentrations to assign exposure due to data availability. Traffic related air pollution (TRAP) is known to exacerbate existing respiratory diseases. Several studies have validated distance to roads as proxies for TRAP^33–35^ in particular around 200 m from major roads. Our findings would be consistent with these other studies. However, we note that in North Carolina, the location of over 94% of the subjects, the mean annual average daily traffic (AADT) on major roads in 2013 was just above 13,000 vehicles per day, whereas the mean AADT on other small roads where traffic counts were measured was ~3,300 vehicles per day^36^. Thus, we chose to include only major roads, anticipating that the lower traffic volume on smaller roads would only contribute to background exposure to traffic-related air pollution. Although we used residential location to estimate the effect traffic-related air pollution exposure has on asthma exacerbations, we did not have information on the length of time an individual has resided at the address provided, nor the amount of time they spend at that residential address on a daily, weekly, or monthly basis.

Further, our pathway approach of examining responder types in conjunction with environmental exposures presents challenges of sample size and power. Although the model controlled for possible confounders (e.g., race, sex, income, BMI, smoking status), there could be systematic differences between individuals within <250 m and ≥250 m, such as stressful life events and noise levels, that our analysis does not account for. We did not consider land use near residences, although land use may affect asthma symptoms^37^. Finally, data obtained from the questionnaire (e.g., race, sex, income, asthma exacerbation) is self-reported, with associated limitations. However, in a separate validation of EPR self-reported data in approximately 1,000 individuals, we found a 94% agreement rate between self-reported and on-site medical history and physical examination results (unpublished results), supporting good reliability of our data.

Despite limitations, our study provides important insights about the relationships between genetic profile, air pollution, and asthma exacerbations. Individual residential addresses allowed us to estimate residential proximity (air pollution exposure) at the individual level instead of in aggregate over a spatial unit (e.g., block group, county). Using distance to roadway to estimate exposure allowed us to include individuals who do not reside near an air quality monitor, which tend to be located in more urban areas^38,39^, effectively limiting the geographic scope and increasing exposure measurement error. Our findings suggest that traffic-related air pollution exposure and specific genotype combinations may affect likelihood of asthma diagnosis and exacerbations, including serious exacerbations (e.g., ER visits). Further studies need to be done to show if avoidance of major roadways based on responder status will decrease asthma exacerbations. However, short-term associations between primary pollutants from traffic sources and pediatric asthma ER visits has been noted^40^. This work supports that targeted association analysis of specific environmentally responsive pathways at a population level may allow the detection of pathway-environment interactions, which underlie asthma development and pathogenesis. Personalized predictions for the activity of a complex disease will require an amalgamation of extensive genotype and environmental data, an era we are approaching with the advent of less expensive whole genome sequencing and deep biomonitoring. Ultimately, multiple such associations may be compiled into a predictive algorithm that can be utilized for individualized asthma management.

## Methods

### Asthma diagnosis and exacerbations

The Environmental Polymorphisms Registry (EPR)^41^ is a repository of DNA samples from over 18,000 individuals. The EPR database contains individual-level self-reported data from 8,843 individuals on physician-diagnosed asthma and presence of asthma exacerbations. Asthma exacerbations included asthma-related activity limitations and sleeplessness in the previous 14-days and asthma-related ER visits in the previous 12-months. Asthma diagnosis and exacerbations were categorized as present/absent.

### DNA genotyping

Blood samples were collected from 2,996 EPR participants for whom health data were available, and total genomic DNA was isolated using Qiagen’s Autopure LS system. DNA was genotyped for four genes in the TLR4 pathway. Samples were genotyped using Illumina multiplexing and/or TaqMan SNP Genotyping Assays (Applied Biosystems): rs8177374, *TIRAP*; rs4986791, *TLR4*; rs2569190, *CD14*; and rs1800629, *TNFa*. Our analyses defined and used individuals with one (heterozygous) or both (homozygous minor) copies of the minor allele as carriers of the SNP. Individuals with both major alleles are WT. Based on genotyping results, subjects were divided into three responder-types:

1. Hyper-responders: [carriers of *CD14* and *TNFa* minor SNPs] and [WT for *TLR4* and *TIRAP* SNPs];
2. Hypo-responders: [carriers of *TLR4* and/or *TIRAP* minor SNPs] and [WT for *CD14* and *TNFa* SNPs];
3. Neither-responders: All others.

Hyper- and hypo-responders were anticipated to have over- and under-reactive responses to air pollution exposure, respectively. Neither-responders were expected to have intermediate responses to pollution.

### Exposure: proximity to a major road

For the 2,996 genotyped individuals, geocoding (assignment of latitude and longitude based on residential address) was performed based on the contact address provided, using ArcGIS 10 software. Of genotyped subjects, 2,830 (94.5%) were successfully geocoded. Individuals with missing covariate data (n = 146) or responder type (n = 16) were removed from the overall analysis dataset, for a sample size of 2,668 individuals. Geocoded individual residential addresses were overlaid with the Streetmap Premium 2014^42^ streets layer. We determined the linear distance between geocoded residential address and the centerline of the nearest major road. The nearest major road included primary [major highways with and without limited access (freeways)] and secondary (primarily state and county highways) roads, based on the Topologically Integrated Geographic Encoding and Referencing database^22^.

Previous studies of asthma have used road proximity as a proxy for traffic-related air pollution exposure^43–47^, but have not categorized distance to road consistently. Data suggest that air pollution levels are elevated near major roads, with pollution levels returning to background concentrations at approximately 300 m^48^. Individuals residing closer to a major road are expected to have systematically higher exposures to traffic-related air pollution than individuals farther from a major road. Thus, we dichotomized distance from each geocoded address to the centerline of the nearest primary or secondary road into <250 m and ≥250 m.

### Statistical analysis

#### Individual SNP genotype associations with asthma diagnosis and exacerbations

Cohort statistics, numbers and percentages, were determined for each SNP for individuals with and without asthma, including sex, race, ethnicity, smoker status, BMI, and annual household income. We performed t-tests on age at asthma diagnosis for each SNP, comparing WT to carrier.

In the subset of participants with asthma, we examined the prevalence of asthma-related exacerbations including activity limitations and sleeplessness during the previous 14-days and asthma-related ER visits in the previous 12-months. Unadjusted odds ratios were generated from logistic regression analyses independently for each SNP. Logistic regression was performed for each SNP with adjustments for sex, race, smoking status, BMI, and income.

Smoking status was operationalized as a binary variable. Individuals who reported previously or currently smoking >100 cigarettes in their lifetime were classified as ever smokers, while those who did not report previous or current tobacco smoking were classified as never smokers. Underweight was classified as a BMI of <18.5, normal weight was a BMI of 18.5 to <25, overweight was a BMI of 25 to <30 and obese was a BMI of 30 or higher. We collapsed income into four categories (<$20,000; $20,000–$39,999; $40,000–$59,999; and ≥$60,000). Due to small cell sizes in the Asian, Native American, Native Hawaiian/Pacific Islander, multiple races, and unknown/not reported race categories, these individuals (n = 144) were combined into an “other races” category.

Statistical analyses were performed using MedCalc for Windows (MedCalc Software, Ostend, Belgium), SAS versions 9.3 and 9.4 (Cary, NC), and R 3.2.2^49^.

#### Responder type, distance to road, and asthma diagnosis and exacerbations

We performed descriptive analyses using proportions for categorical variables and means and medians for continuous variables (e.g., distance to major road prior to categorization into <250 m and ≥250 m). Tests for homogeneity in individual characteristics across the three responder-types and two distance to roadway groups were evaluated using χ^2^ contingency table statistics for categorical variables. To evaluate our hypothesis that asthma diagnosis and exacerbations are associated with proximity to a major road and responder type, we examined asthma diagnosis using logistic regression to model presence/absence of an asthma diagnosis. We also examined presence/absence of asthma exacerbations using self-reported data on asthma-related ER visits, sleeplessness, and activity limitations, in both unadjusted and adjusted models. A third model specification was fit to examine our hypothesis of an interaction between responder type and distance to roadway on asthma diagnosis and exacerbations.

Adjusted models controlled for covariates available in the EPR database and suspected to be potential confounders in the existing literature^50–53^: sex, race, smoking status, BMI, and income.

We used likelihood ratio tests to compare nested models, testing the hypothesis that adjusted models are an improved fit over unadjusted models. As a sensitivity analysis, we re-analyzed data using the same methodology but included tertiary roads, classifying proximity to road as <250 m and ≥250 m from the nearest primary, secondary, or tertiary road. We also applied different classifications for near versus far distance from road, including 300 m, 400 m, and 500 m, in addition to the 250 m presented in the main analysis. A 2-sided 95% significance level was used for all statistical inference (α < 0.05), unless stated otherwise.

This research was conducted under NHGRI, NIH Institutional Review Board approved protocol #14-E-0053 and all participants have provided written informed consent. All methods were performed in accordance with the relevant guidelines and regulations.

## Electronic supplementary material

Supplemental Information
