# Supplementary material for: Toll-like Receptor 4 Pathway Polymorphisms Interact with Pollution to Influence Asthma Diagnosis and Severity
Source: Sci Rep. 2018 Aug 23;8:12713. doi: 10.1038/s41598-018-30865-0 (PMC6107668; doi:10.1038/s41598-018-30865-0)
Supplement: Supplementary file 1 — Supplemental Information [file 41598_2018_30865_MOESM1_ESM.docx]

**Supplemental Information**

**Toll-like Receptor 4 Pathway Polymorphisms Interact with Pollution to Influence Asthma Diagnosis and Severity**

Shepherd H. Schurman, Mercedes A. Bravo, Cynthia L. Innes, W. Braxton Jackson II, John A. McGrath, Marie Lynn Miranda, and Stavros Garantziotis

**Table of Contents**

**Table S1.** Asthma-Related Exacerbations in All Geocoded Participants with Asthma by TNFα Genotype

**Table S2.** Asthma-Related Exacerbations in All Geocoded Participants with Asthma by CD14 Genotype

**Table S3.** Asthma-Related Exacerbations in All Geocoded Participants with Asthma by TLR4 Genotype

**Table S4.** Asthma-Related Exacerbations in All Geocoded Participants with Asthma by TIRAP Genotype

**Table S5.** Prevalence of Susceptibility to Innate Immune Stimuli by Physician Diagnosis of Asthma, Genotyped EPR Questionnaire Participants

**Table S6.** Mean Age of Asthma Diagnosis by Susceptibility to Innate Immune Stimuli, Genotyped EPR Questionnaire Participants

**Table S7.** Distribution of Asthma-Related Exacerbations during past 14 Days by Susceptibility to Innate Immune Stimuli

**Table S8.** Prevalence of Asthma-Related Exacerbation during past 12 Months by Susceptibility to Innate Immune Stimuli

**Table S9.** Distribution of covariates by responder type

**Table S10.** Distribution of covariates by distance to primary or secondary road

**Table S11.** Sensitivity analysis: Asthma diagnosis, responder status, and distance to road

**Table S12.** Sensitivity analysis: Asthma exacerbations, responder status, and distance to road

**Table S13.** Distribution of covariates by distance to primary, secondary, or tertiary road

**Table S14.** Sensitivity analysis: Asthma diagnosis, responder status, and distance to road for the interaction model

**Table S15.** Sensitivity analysis: Asthma exacerbations, responder status, and 300m distance to road

**Table S16.** Sensitivity analysis: Asthma exacerbations, responder status, and 400m distance to road

**Table S17.** Sensitivity analysis: Asthma exacerbations, responder status, and 500m distance to road

| **Table S1. Asthma-Related Exacerbations in All Geocoded Participants with Asthma by TNFα Genotype** | | | | | | | | | | | |  |  |
| --- | --- | --- | --- | --- | --- | --- | --- | --- | --- | --- | --- | --- | --- |
|  |  |  |  |  |  |  | |  | |  | |  |  |
|  | | **Major** | | **Het/Minor** | | **Logistic Regression** | | | | | | | |
| **Asthma-Related Exacerbations^1^** | | **N^2^** | **%** | **N^2^** | **%** | **Unadjusted Odds Ratio** | | **95% CI** | | **Adjusted Odds Ratio^3^** | | **95% CI** | |
| ***Days with Activity***  ***Limitations*** | |  |  |  |  |  | |  | |  | |  | |
| None | | 422 | 64.6% | 165 | 62.3% |  | |  | |  | |  | |
| 1 to 14 | | 231 | 35.4% | 100 | 37.7% | 1.11 | | 0.82 to 1.49 | | 1.19 | | 0.85 to 1.65 | |
| ***Days with***  ***Sleeplessness*** | |  |  |  |  |  | |  | |  | |  | |
| None | | 504 | 76.8% | 204 | 77.6% |  | |  | |  | |  | |
| 1 to 14 | | 152 | 23.2% | 59 | 22.4% | 0.96 | | 0.68 to 1.35 | | 0.94 | | 0.63 to 1.39 | |
| ***Asthma-related ER***  ***visit in past 12***  ***months*** | |  |  |  |  |  | |  | |  | |  | |
| No | | 590 | 87.4% | 242 | 87.7% |  | |  | |  | |  | |
| Yes | | 85 | 12.6% | 34 | 12.3% | 0.98 | | 0.64 to 1.49 | | 0.97 | | 0.61 to 1.55 | |
| ***Any Exacerbation:***  ***Activity Limitations,***  ***Sleeplessness, or***  ***ER visits*** | |  |  |  |  |  | |  | |  | |  | |
| No | | 406 | 59.7% | 157 | 56.5% |  | |  | |  | |  | |
| Yes | | 274 | 40.3% | 121 | 43.5% | 1.14 | | 0.86 to 1.51 | | 1.20 | | 0.88 to 1.65 | |

| ^1^The total number of respondents to asthma outcomes differs between categories due to item non-response. | | | | | | |
| --- | --- | --- | --- | --- | --- | --- |
| ^2^Total number of respondents for given response |  |  |  |  |  |  |
| ^3^Logistic regression was performed with covariates of gender, race, smoking status, BMI and annual household income. | | | | | | |

| **Table S2. Asthma-Related Exacerbations in All Geocoded Participants with Asthma by**  **CD14 Genotype** | | | | | | | | | | | | | | | | | | | | |  |
| --- | --- | --- | --- | --- | --- | --- | --- | --- | --- | --- | --- | --- | --- | --- | --- | --- | --- | --- | --- | --- | --- |
|  | |  | | |  | |  | | |  | |  | | |  | |  | | |  |  |
|  | **Major** | | | | | **Het/Minor** | | | | | **Logistic Regression** | | | | | | | | | | |
| **Asthma-Related Exacerbations^1^** | **N^2^** | | | **%** | | **N^2^** | | | **%** | | **Unadjusted Odds Ratio** | | | **95% CI** | | **Adjusted Odds Ratio^3^** | | **95% CI** | | | |
| ***Days with Activity***  ***Limitations*** |  | |  | | |  | |  | | |  | |  | | |  | | |  | | |
| None | 165 | | 63.2% | | | 422 | | 64.2% | | |  | |  | | |  | | |  | | |
| 1 to 14 | 96 | | 36.8% | | | 235 | | 35.8% | | | 0.96 | | 0.71 to 1.29 | | | 1.16 | | | 0.82 to 1.64 | | |
| ***Days with***  ***Sleeplessness*** |  | |  | | |  | |  | | |  | |  | | |  | | |  | | |
| None | 199 | | 76.5% | | | 509 | | 77.2% | | |  | |  | | |  | | |  | | |
| 1 to 14 | 61 | | 23.5% | | | 150 | | 22.8% | | | 0.96 | | 0.68 to 1.35 | | | 1.45 | | | 0.96 to 2.19 | | |
| ***Asthma-related ER***  ***visit in past 12***  ***months*** |  | |  | | |  | |  | | |  | |  | | |  | | |  | | |
| No | 239 | | 87.5% | | | 593 | | 87.5% | | |  | |  | | |  | | |  | | |
| Yes | 34 | | 12.5% | | | 85 | | 12.5% | | | 1.01 | | 0.66 to 1.54 | | | 1.53 | | | 0.93 to 2.52 | | |
| ***Any Exacerbation:***  ***Activity Limitations,***  ***Sleeplessness, or***  ***ER visits*** |  | |  | | |  | |  | | |  | |  | | |  | | |  | | |
| No | 163 | | 59.3% | | | 400 | | 58.6% | | |  | |  | | |  | | |  | | |
| Yes | 112 | | 40.7% | | | 283 | | 41.4% | | | 1.03 | | 0.77 to 1.37 | | | 1.28 | | | 0.91 to 1.78 | | |

| ^1^The total number of respondents to asthma outcomes differs between categories due to item non-response. | | | | | | |
| --- | --- | --- | --- | --- | --- | --- |
| ^2^Total number of respondents for given response |  |  |  |  |  |  |
| ^3^Logistic regression was performed with covariates of gender, race, smoking status, BMI and annual household income. | | | | | | |

| **Table S3. Asthma-Related Exacerbations in All Geocoded Participants with Asthma by**  **TLR4 Genotype** | | | | | | | | | | | | | | | | | | | | |  |
| --- | --- | --- | --- | --- | --- | --- | --- | --- | --- | --- | --- | --- | --- | --- | --- | --- | --- | --- | --- | --- | --- |
|  | |  | | |  | |  | | |  | |  | | |  | |  | | |  |  |
|  | **Major** | | | | | **Het/Minor** | | | | | **Logistic Regression** | | | | | | | | | | |
| **Asthma-Related Exacerbations^1^** | **N^2^** | | | **%** | | **N^2^** | | | **%** | | **Unadjusted Odds Ratio** | | | **95% CI** | | **Adjusted Odds Ratio^3^** | | **95% CI** | | | |
| ***Days with Activity***  ***Limitations*** |  | |  | | |  | |  | | |  | |  | | |  | | |  | | |
| None | 524 | | 63.2% | | | 63 | | 70.8% | | |  | |  | | |  | | |  | | |
| 1 to 14 | 305 | | 36.8% | | | 26 | | 29.2% | | | 0.71 | | 0.44 to 1.14 | | | 0.76 | | | 0.45 to 1.29 | | |
| ***Days with***  ***Sleeplessness*** |  | |  | | |  | |  | | |  | |  | | |  | | |  | | |
| None | 633 | | 76.2% | | | 75 | | 85.2% | | |  | |  | | |  | | |  | | |
| 1 to 14 | 198 | | 23.8% | | | 13 | | 14.8% | | | 0.55 | | 0.30 to 1.02 | | | 0.79 | | | 0.41 to 1.54 | | |
| ***Asthma-related ER***  ***visit in past 12***  ***months*** |  | |  | | |  | |  | | |  | |  | | |  | | |  | | |
| No | 747 | | 86.8% | | | 85 | | 94.4% | | |  | |  | | |  | | |  | | |
| Yes | 114 | | 13.2% | | | 5 | | 5.6% | | | **0.39** | | **0.15 to 0.97** | | | 0.55 | | | 0.21 to 1.44 | | |
| ***Any Exacerbation:***  ***Activity Limitations,***  ***Sleeplessness, or***  ***ER visits*** |  | |  | | |  | |  | | |  | |  | | |  | | |  | | |
| No | 502 | | 57.9% | | | 61 | | 67.0% | | |  | |  | | |  | | |  | | |
| Yes | 365 | | 42.1% | | | 30 | | 33.0% | | | 0.68 | | 0.43 to 1.07 | | | 0.79 | | | 0.48 to 1.31 | | |

| ^1^The total number of respondents to asthma outcomes differs between categories due to item non-response. | | | | | | |
| --- | --- | --- | --- | --- | --- | --- |
| ^2^Total number of respondents for given response |  |  |  |  |  |  |
| ^3^Logistic regression was performed with covariates of gender, race, smoking status, BMI and annual household income. | | | | | | |

| **Table S4. Asthma-Related Exacerbations in All Geocoded Participants with Asthma by**  **TIRAP Genotype** | | | | | | | | | | | | | | | | | | | | |  |
| --- | --- | --- | --- | --- | --- | --- | --- | --- | --- | --- | --- | --- | --- | --- | --- | --- | --- | --- | --- | --- | --- |
|  | |  | | |  | |  | |  | | |  | | |  |  | |  | | |  |
|  | **Major** | | | | | **Het/Minor** | | | | | **Logistic Regression** | | | | | | | | | | |
| **Asthma-Related Exacerbations^1^** | **N^2^** | | | **%** | | **N^2^** | | | | **%** | **Unadjusted Odds Ratio** | | | **95% CI** | | | **Adjusted Odds Ratio^3^** | | **95% CI** | | |
| ***Days with Activity***  ***Limitations*** |  | |  | | |  | |  | | |  | |  | | | |  | | |  | |
| None | 436 | | 62.3% | | | 151 | | 69.3% | | |  | |  | | | |  | | |  | |
| 1 to 14 | 264 | | 37.7% | | | 67 | | 30.7% | | | 0.73 | | 0.53 to 1.02 | | | | 0.87 | | | 0.60 to 1.26 | |
| ***Days with***  ***Sleeplessness*** |  | |  | | |  | |  | | |  | |  | | | |  | | |  | |
| None | 535 | | 76.2% | | | 173 | | 79.7% | | |  | |  | | | |  | | |  | |
| 1 to 14 | 167 | | 23.8% | | | 44 | | 20.3% | | | 0.81 | | 0.56 to 1.18 | | | | 1.22 | | | 0.79 to 1.90 | |
| ***Asthma-related ER***  ***visit in past 12***  ***months*** |  | |  | | |  | |  | | |  | |  | | | |  | | |  | |
| No | 633 | | 87.6% | | | 199 | | 87.3% | | |  | |  | | | |  | | |  | |
| Yes | 90 | | 12.4% | | | 29 | | 12.7% | | | 1.03 | | 0.66 to 1.60 | | | | 1.53 | | | 0.90 to 2.60 | |
| ***Any Exacerbation:***  ***Activity Limitations,***  ***Sleeplessness, or***  ***ER visits*** |  | |  | | |  | |  | | |  | |  | | | |  | | |  | |
| No | 422 | | 57.8% | | | 141 | | 61.8% | | |  | |  | | | |  | | |  | |
| Yes | 308 | | 42.2% | | | 87 | | 38.2% | | | 0.85 | | 0.62 to 1.15 | | | | 1.06 | | | 0.74 to 1.50 | |

| ^1^The total number of respondents to asthma outcomes differs between categories due to item non-response. | | | | | | |
| --- | --- | --- | --- | --- | --- | --- |
| ^2^Total number of respondents for given response |  |  |  |  |  |  |
| ^3^Logistic regression was performed with covariates of gender, race, smoking status, BMI and annual household income. | | | | | | |

| **Table S5. Prevalence of Susceptibility to Innate Immune Stimuli by Physician Diagnosis of Asthma, Genotyped EPR Questionnaire Participants** | | | | |
| --- | --- | --- | --- | --- |
|  |  |  |  |  |
|  |  |  |  |  |
|  |  |  |  |  |
|  | **MD Diagnosis of Asthma^1^** | | | |
|  | **Yes** | | **No** | |
| **Susceptibility to Innate Immune Stimuli** | **N** | **%** | **N** | **%** |
| Hyper-responder | 137 | 14.2 | 232 | 13.3 |
| Hypo-responder | 44 | 4.6 | 88 | 5.1 |
| Neither | 782 | 81.2 | 1,421 | 81.6 |
| Total | 963 | 100.0 | 1,741 | 100.0 |
| ^1.^ Non-asthmatics includes 53 participants who did not answer question on diagnosed asthma and were coded as “No.” Excluding these participants would not markedly change responder status percentages (13.0% would be Hyper-responders, 5.2% would be Hypo-responders, and 81.8% would be Neither). | | | | |
| Note: A Chi Square test indicated that there was no significant variation in the distribution of responder class among those with and without asthma (X^2^ = 0.68, df = 2, *p* = 0.71). | | | | |
|  |  |  |  |  |
|  |  |  |  |  |

| **Table S6. Mean Age of Asthma Diagnosis by Susceptibility to Innate Immune Stimuli, Genotyped EPR Questionnaire Participants** | | | | | |
| --- | --- | --- | --- | --- | --- |
|  |  |  |  |  |  |
|  |  |  |  |  |  |
| **Mean Age at Diagnosis** | | | |  |  |
| **Susceptibility to Innate Immune Stimuli** | **N** | **Mean (years)** | **SD** |  |  |
| Hyper-responder | 133 | 22.4 | 17.7 |  |  |
| Hypo-responder | 42 | 26.3 | 20.2 |  |  |
| Neither | 748 | 23.2 | 18.4 |  |  |
| Total | 923 | 23.2 | 18.4 |  |  |
|  |  |  |  |  |  |
| Note: A comparison of means t-test showed no significant association of mean age at diagnosis with responder status. | | | |  |  |
|  |  |  |  |  |  |
|  |  |  |  |  |  |
| Hypo-responder vs. Hyper responder | | *p*=0.23 |  |  |  |
| Hypo-responder vs. Neither | | *p*=0.29 |  |  |  |
| Hyper-responder vs. Neither | | *p*=0.66 |  |  |  |

| **Table S7. Distribution of Asthma-Related Exacerbations during past 14 Days by Susceptibility to Innate Immune Stimuli** | | | | | | | | |
| --- | --- | --- | --- | --- | --- | --- | --- | --- |
|  |  |  |  |  |  |  |  |  |
|  |  |  |  |  |  |  |  |  |
|  |  |  |  |  |  |  |  |  |
|  | **Susceptibility to Innate Immune Stimuli** | | | | | | | |
|  | **Hyper-responder** | | **Hypo-responder** | | **Neither** | | **Total** | |
| **Frequency During Past 14 Days** | **N** | **%** | **N** | **%** | **N** | **%** | **N** | **%** |
| Activity Limitations |  |  |  |  |  |  |  |  |
| None | 79 | 59.9 | 29 | 72.5 | 479 | 64.2 | 587 | 63.9 |
| 1 to 14 | 53 | 40.1 | 11 | 27.5 | 267 | 35.8 | 331 | 36.1 |
| Sleepless Nights |  |  |  |  |  |  |  |  |
| None | 103 | 78.0 | 35 | 87.5 | 570 | 76.3 | 708 | 77.0 |
| 1 to 14 | 29 | 22.0 | 5 | 12.5 | 177 | 23.7 | 211 | 23.0 |
|  | | | | |  |  |  |  |
| Hypo-responder vs. Hyper-responder for: | | | |  |  |  |  |  |
| Sleepless Nights: Any (1-14days) vs. None *p*=0.19 | | | | |  |  |  |  |
| Activity Limitations: Any (1-14days) vs. None *p*=0.15 | | | | |  |  |  |  |

| **Table S8. Prevalence of Asthma-Related Exacerbation during past 12 Months by Susceptibility to Innate Immune Stimuli** | | | | | | | | | | | | |  |
| --- | --- | --- | --- | --- | --- | --- | --- | --- | --- | --- | --- | --- | --- |
|  |  |  |  |  |  |  |  |  |  |  |  |  |  |
|  |  |  |  |  |  |  |  |  |  |  |  |  |  |
|  | **Susceptibility to Innate Immune Stimuli** | | | | | | | | | | | |  |
|  | **Hyper-responder** | | | **Hypo-responder** | | | **Neither** | | |  | **Total** |  |  |
| **Asthma-Related Exacerbation^1^** | **N^2^** | **n^3^** | **%** | **N^2^** | **n^3^** | **%** | **N^2^** | **n^3^** | **%** | **N^2^** | **n^3^** | **%** |  |
| Asthma-Related ER visit in past 12 months | 136 | 15 | 11.0 | 44 | 3 | 6.8 | 771 | 101 | 13.1 | 951 | 119 | 12.5 |  |
| ^1.^ The total number of respondents to asthma exacerbation over 12 months differs with 14 days due to item non-response or participant change in asthma status in the last 12 months. | | | | | | | | | | | | | |
|  |  |  |  |  |  |  |  |  |  |  |  |  |  |
| ^2.^Total number of respondents | | | | | | | | | |  |  |  |  |
| ^3.^Total number answering yes | | | | | | | | | |  |  |  |  |
|  | | | | |  |  |  |  |  |  |  |  |  |
| Hypo-responder vs. Hyper-responder for | | | |  |  |  |  |  |  |  |  |  |  |
| Current Asthma: *p*=0.81 | |  |  |  |  |  |  |  |  |  |  |  |  |
| Asthma-Related ER visit in past 12 months: *p*=0.42 | | | | |  |  |  |  |  |  |  |  |  |

| **Table S9. Distribution of covariates by responder type** | | | |
| --- | --- | --- | --- |
|  | **Responder type** | | |
|  | Neither  N (%) | Hyper  N (%) | Hypo  N (%) |
| **All observations**^a^ (n=2,668) | 2,175 (81.5) | 363 (13.6) | 130 (4.9) |
| *Sex*^b^ | | | |
| Female | 1,375 (63.2) | 213 (58.7) | 75 (57.7) |
| Male | 800 (36.8) | 150 (41.3) | 55 (42.3) |
| *Race* | | | |
| BAA | 687 (31.6) | 101 (27.8) | 19 (14.6) |
| White | 1,380 (63.4) | 243 (66.9) | 94 (72.3) |
| Other | 108 (5.0) | 19 (5.2) | 17 (13.1) |
| *Ethnicity* |  |  |  |
| Hispanic | 121 (5.6) | 17 (4.7) | 9 (6.9) |
| Non-Hispanic | 2,007 (92.3) | 336 (92.6) | 119 (91.5) |
| Unknown/Not reported | 47 (2.2) | 10 (2.7) | 2 (1.5) |
| *Distance to nearest primary or secondary road* | | | |
| < 250m | 164 (7.5) | 26 (7.3) | 10 (7.7) |
| >250m | 2,011 (92.5) | 337 (92.7) | 120 (92.3) |
| *Distance to nearest primary, secondary, or tertiary road* | | | |
| < 250m | 926 (42.6) | 180 (49.6) | 52 (40.0) |
| >250m | 1,249 (57.4) | 183 (50.4) | 78 (60.0) |
| *Body mass index (BMI)* |  |  |  |
| Normal or underweight | 586 (26.9) | 103 (28.4) | 38 (29.2) |
| Overweight or obese | 1,589 (73.1) | 260 (71.6) | 92 (70.8) |
| *Income ($/year)* | | | |
| < $20,000 | 370 (17.0) | 79 (21.8) | 19 (14.6) |
| $20,000 - $39,999 | 491 (22.6) | 63 (17.4) | 27 (20.8) |
| $40,000 - $59,999 | 423 (19.4) | 58 (16.0) | 21 (16.2) |
| > $60,000 | 891 (41.0) | 163 (44.9) | 63 (48.4) |
| *Smoker* | 950 (43.7) | 162 (44.6) | 59 (45.3) |
| ^a^ The total number of geocoded individuals in the initial dataset was n = 2,830. Individuals with missing responder type (n = 16) or missing covariates (n = 146) were excluded from the dataset (final n = 2,668). | | | |

^b^ Percentages presented in this row and following rows are percentages based on the column total.

| **Table S10. Distribution of covariates by distance to primary or secondary road** | | |
| --- | --- | --- |
|  | **Distance to road** | |
|  | < 250m  N (%) | > 250m  N (%) |
| **All observations**^a^ (n=2,668) | 200 (7.5) | 2,468 (92.5) |
| *Sex*^b^ | | |
| Female | 134 (67.0) | 1,529 (62.0) |
| Male | 66 (33.0) | 939 (38.0) |
| *Race* | | |
| BAA | 72 (36.0) | 735 (29.8) |
| White | 118 (59.0) | 1,599 (64.8) |
| Other | 10 (5.0) | 134 (5.42) |
| *Ethnicity* |  |  |
| Hispanic | 15 (7.5) | 132 (5.4) |
| Non-Hispanic | 179 (89.5) | 2,283 (92.5) |
| Unknown/Not reported | 6 (3.0) | 53 (2.1) |
| *Responder type* | | |
| Neither | 164 (82.0) | 2,011 (81.4) |
| Hyper | 26 (13.0) | 337 (13.7) |
| Hypo | 10 (5.0) | 120 (4.9) |
| *Body mass index (BMI)* |  |  |
| Normal or underweight | 68 (34.0) | 659 (26.7) |
| Overweight or obese | 132 (66.0) | 1,809 (73.3) |
| *Income ($/year)* | | |
| < $20,000 | 51 (25.5) | 417 (16.9) |
| $20,000 - $39,999 | 47 (23.5) | 534 (21.6) |
| $40,000 - $59,999 | 36 (18.0) | 466 (18.9) |
| > $60,000 | 66 (33.0) | 1,051 (42.6) |
| *Smoker* | 80 (40.0) | 1,091 (44.2) |
| ^a^ The total number of geocoded individuals in the initial dataset was n = 2,830. Individuals with missing responder type (n = 16) or missing covariates (n = 146) were excluded from the dataset (final n = 2,668).  ^b^ Percentages presented in this row and following rows are percentages based on the column total (i.e., denominator = 205 for <250m and 2,499 for >250m). | | |

| **Table S11. Sensitivity analysis: Asthma diagnosis, responder status, and distance to road**^a^ | | | |
| --- | --- | --- | --- |
|  | Unadjusted^b^  OR (95% CI) | Adjusted^c^  OR (95% CI) | Interaction^d^  OR (95% CI) |
| Reference group – No asthma^e^ | 1.00 (reference) | 1.00 (reference) | 1.00 (reference) |
| Reference group – Asthma^f^ | **0.51 (0.46, 0.57)*** | **0.71 (0.57, 0.88)*** | **0.69 (0.56, 0.87)*** |
| < 250 m | **1.18 (1.01, 1.39)*** | **1.20 (1.01, 1.42)*** | **1.24 (1.03, 1.49)*** |
| Hyper responder | 1.06 (0.84, 1.33) | 1.06 (0.83, 1.35) | 1.11 (0.79, 1.55) |
| Hypo responder | 0.91 (0.52, 1.31) | 0.84 (0.57, 1.23) | 1.02 (0.62, 1.66) |
| Ever smoker | -- | 0.99 (0.83, 1.17) | 0.99 (0.83, 1.17) |
| Male | -- | **0.41 (0.34, 0.49)*** | **0.41 (0.34, 0.49)*** |
| African American | -- | **0.56 (0.45, 0.68)*** | **0.55 (0.45, 0.67)*** |
| Other races | -- | *1.38 (0.97, 1.97)^+^* | *1.38 (0.97, 1.96)^+^* |
| Overweight or obese |  | **1.11 (0.92, 1.34)** | **1.11 (0.92, 1.34)** |
| Income < $20,000 | -- | **1.58 (1.21, 1.96)*** | **1.54 (1.21, 1.96)*** |
| Income $20,000-$39,999 | -- | 0.94 (0.75, 1.19) | 0.94 (0.75, 1.17) |
| Income $40,000-$59,999 | -- | 0.95 (0.75, 1.19) | 0.95 (0.75, 1.20) |
| < 250 m distance*Hyper | -- | -- | 0.90 (0.56, 1.46) |
| < 250 m distance*Hypo | -- | -- | 0.61 (0.27, 1.35) |
| ^a^ Distance to roadway classified as < 250 m and > 250 m from nearest primary, secondary, or tertiary road  ^b^ Unadjusted models included only responder and distance category  ^c^ Adjusted models included responder, distance category, sex, race, smoking status, body mass index, and income category.  ^d^ Interaction models included all covariates in adjusted models in addition to an interaction term for distance and responder type.  ^e^ Reference group is neither responder, > 250 m from road, Caucasian, female, nonsmoker, normal or underweight body mass index, income > $60,000.  ^f^ OR reported in this row indicate the odds of a member of the reference group having asthma.  *Indicates significance at *p* < 0.05  ^+^ Indicates *p* < 0.10 | | | |

| **Table S12. Sensitivity analysis: Asthma exacerbations, responder status, and distance to road**^a,b^ | | | | |
| --- | --- | --- | --- | --- |
|  | Activity limitations  OR (95% CI) | Sleeplessness  OR (95% CI) | Emergency room visits  OR (95% CI) | Any exacerbations  OR (95% CI) |
| Reference group:  Asthma diagnosis, no exacerbation^c^ | 1.00 (reference) | 1.00 (reference) | 1.00 (reference) | 1.00 (reference) |
| Reference group^d^ | **0.25 (0.17, 0.37)*** | **0.09 (0.05, 0.14)*** | **0.04 (0.02, 0.08)*** | **0.34 (0.23, 0.49)*** |
| < 250 m distance | 0.86 (0.64, 1.16) | 0.88 (0.62, 1.25) | 0.77 (0.5-, 1.19) | 0.81 (0.61, 1.08) |
| Hyper responder | 1.29 (0.86, 1.93) | 0.93 (0.56, 1.50) | 0.88 (0.46, 1.58) | 1.23 (0.83, 1.83) |
| Hypo responder | **0.45 (0.20, 0.94)^*^** | **0.36 (0.12, 0.90)*** | 0.46 (0.11, 1.38) | **0.48 (0.23, 0.96)*** |
| Smoker | 1.27 (0.95, 1.69)^+^ | **1.72 (1.23, 2.43)*** | 1.35 (0.89, 2.07) | 1.22 (0.92, 1.62) |
| Male | **0.64 (0.45, 0.89)*** | **0.38 (0.24, 0.59)*** | 0.78 (0.46, 1.28) | **0.56 (0.41, 0.79)*** |
| African American | 0.878 (0.54, 1.11) | **1.59 (1.08, 2.34)*** | **1.75 (1.10, 2.78)*** | 0.98 (0.69, 1.38) |
| Other races | 1.20 (0.68, 2.08) | 1.14 (0.56, 2.21) | 0.96 (0.35, 2.24) | 1.42 (0.83, 2.44) |
| Overweight or obese | **1.45 (1.05, 2.02)*** | **1.77 (1.18, 2.68)** | **1.71 (1.03, 2.94)*** | **1.59 (1.16, 2.18)*** |
| Income < $20,000 | **3.85 (2.59, 5.73)*** | **4.65 (2.95, 7.41)*** | **5.28 (3.00, 9.55)*** | **3.94 (2.67, 5.87)*** |
| Income $20,000-$39,999 | **2.61 (1.76, 3.88)*** | **2.36 (1.47, 3.790)*** | **2.08 (1.11, 3.90)*** | **2.10 (1.44, 3.07)*** |
| Income $40,000-$59,999 | 1.37 (0.89, 2.09) | 1.25 (0.71, 2.15) | 1.11 (0.50, 2.32) | 1.24 (0.82, 1.84) |
| ^a^ Distance to roadway classified as < 250 m and > 250 m from nearest primary, secondary, or tertiary road  ^b^ Results reported are for adjusted models, which include sex, race, smoking status, body mass index, and income category as covariates  ^c^ Reference group is neither responder, > 250 m from road, Caucasian, female, nonsmoker, income > $60,000  ^d^ OR reported in this row indicate the odds of a member of the reference group having an asthma exacerbation.  *Indicates significance at *p* < 0.05  ^+^ Indicates *p* < 0.10 | | | | |

| **Table S13. Distribution of covariates by distance to primary, secondary, or tertiary road** | | |
| --- | --- | --- |
|  | **Distance to road** | |
|  | < 250m  N (%) | > 250m  N (%) |
| **All observations**^a^ (n=2,668) | 1,158 (43.6) | 1,510 (56.4) |
| *Sex*^b^ | | |
| Female | 737 (63.6) | 926 (61.3) |
| Male | 421 (36.4) | 584 (38.7) |
| *Race* | | |
| BAA | 445 (38.4) | 362 (24.0) |
| White | 642 (55.42) | 1,075 (71.2) |
| Other | 71 (6.13) | 73 (4.8) |
| *Ethnicity* |  |  |
| Hispanic | 57 (4.92) | 90 (6.0) |
| Non-Hispanic | 1,071 (92.5) | 1,391 (92.1) |
| Unknown/Not reported | 30 (2.59) | 29 (1.9) |
| *Responder type* | | |
| Neither | 926 (80.0) | 1,249 (82.7) |
| Hyper | 180 (15.5) | 183 (12.1) |
| Hypo | 52 (4.5) | 78 (5.2) |
| *Body mass index (BMI)* |  |  |
| Normal or underweight | 333 (28.8) | 394 (26.1) |
| Overweight or obese | 825 (71.2) | 1,116 (73.9) |
| *Income ($/year)* | | |
| < $20,000 | 286 (24.7) | 182 (12.1) |
| $20,000 - $39,999 | 269 (23.2) | 312 (20.7) |
| $40,000 - $59,999 | 196 (16.9) | 306 (20.2) |
| > $60,000 | 407 (35.1) | 710 (47.0) |
| *Smoker* | 514 (44.4) | 657 (43.5) |
| \| ^a^ The total number of geocoded individuals in the initial dataset was n = 2,830. Individuals with missing responder type (n = 16) or missing covariates (n = 146) were excluded from the dataset (final n = 2,668).  ^b^ Percentages presented in this row and following rows are percentages based on the column total. \| \| --- \| | | |

| **Table S14. Sensitivity analysis: Asthma diagnosis, responder status, and distance to road for the interaction model**^a^ | | | |
| --- | --- | --- | --- |
|  | 300m  OR (95% CI) | 400m  OR (95% CI) | 500m  OR (95% CI) |
| Reference group – No asthma^e^ | 1.00 (reference) | 1.00 (reference) | 1.00 (reference) |
| Reference group – Asthma^f^ | **0.75 (0.61, 0.92)*** | **0.76 (0.61, 0.95)*** | **0.75 (0.61, 0.93)*** |
| Near road | 1.03 (0.75, 1.41) | 0.98 (0.74, 1.29) | 1.00 (0.78, 1.28) |
| Hyper responder | 1.02 (0.79, 1.30) | 1.01 (0.78, 1.31) | 0.99 (0.76, 1.28) |
| Hypo responder | 0.81 (0.53, 1.22) | 0.77 (0.50, 1.16) | 0.80 (0.52, 1.21) |
| Ever smoker | 0.98 (0.83, 1.17) | 0.98 (0.83, 1.16) | 0.99 (0.83, 1.17) |
| Male | **0.41 (0.35, 0.49)*** | **0.41 (0.34, 0.49)*** | **0.41 (0.34, 0.49)** |
| African American | **0.57 (0.48, 0.69)*** | **0.57 (0.47, 0.70)*** | **0.57 (0.47, 0.70)** |
| Other races | *1.41 (0.99, 2.01)^+^* | *1.41 (0.99, 2.02)^+^* | *1.41 (0.99, 2.00)^+^* |
| Overweight or obese | 1.11 (0.92, 1.34) | 1.11 (0.92, 1.34) | 1.11 (0.92, 1.34) |
| Income < $20,000 | **1.58 (1.24, 2.0-)*** | **1.57 (1.24, 2.01)*** | **1.58 (1.24, 2.01)*** |
| Income $20,000-$39,999 | 0.95 (0.75, 1.19) | 0.94 (0.75, 1.18) | 0.95 (0.75, 1.18) |
| Income $40,000-$59,999 | 0.95 (0.75, 1.19) | 0.95 (0.75, 1.18) | 0.95 (0.75, 1.20) |
| Near road*Hyper | 1.86 (0.82, 4.30*)* | 1.66 (0.80, 3.47) | *1.75 (0.91, 3.37)^+^* |
| Near road*Hypo | 1.40 (0.38, 4.84) | 1.99 (0.63, 6.14) | 1.40 (0.46, 4.01) |
| ^a^ Interaction models included all covariates in adjusted models in addition to an interaction term for distance and responder type.  ^e^ Reference group is neither responder, far from road, Caucasian, female, nonsmoker, normal or underweight body mass index, and income > $60,000.  ^f^ OR reported in this row indicate the odds of a member of the reference group having asthma.  *Indicates significance at *p* < 0.05  ^+^ Indicates *p* < 0.10 | | | |

| **Table S15. Sensitivity analysis: Asthma exacerbations, responder status, and 300m distance to road**^a,b^ | | | | |
| --- | --- | --- | --- | --- |
|  | Activity limitations  OR (95% CI) | Sleeplessness  OR (95% CI) | Emergency room visits  OR (95% CI) | Any exacerbations  OR (95% CI) |
| Reference group:  Asthma diagnosis, no exacerbation^c^ | 1.00 (reference) | 1.00 (reference) | 1.00 (reference) | 1.00 (reference) |
| Reference group^d^ | **0.24 (0.17, 0.35)*** | **0.08 (0.05, 0.14)** | **0.039 (0.020, 0.074)*** | **0.33 (0.23, 0.47)*** |
| < 300 m distance | 0.74 (0.45, 1.19) | 0.78 (0.43, 1.36) | 0.64 (0.28, 1.28) | 0.70 (0.43, 1.11) |
| Hyper responder | 1.29 (0.86, 1.92) | 0.92 (0.55, 1.48) | 0.85 (0.45, 1.53) | 1.22 (0.82, 1.81) |
| Hypo responder | **0.46 (0.21, 0.95)^*^** | **0.36 (0.12, 0.91)*** | 0.47 (0.11, 1.41) | **0.49 (0.24, 0.99)*** |
| Smoker | 1.26 (0.95, 1.69)^+^ | **1.72 (1.23, 2.42)*** | 1.35 (0.89, 2.06) | 1.22 (0.92, 1.62) |
| Male | **0.63 (0.45, 0.89)*** | **0.38 (0.24, 0.59)*** | 0.77 (0.46, 1.26) | **0.56 (0.40, 0.76)*** |
| African American | 0.76 (0.54, 1.08) | **1.57 (1.07, 2.29)*** | **1.68 (1.07, 2.65)*** | 0.95 (0.68, 1.34) |
| Other races | 1.18 (0.67, 2.03) | 1.12 (0.54, 2.16) | 0.91 (0.33, 2.12) | 1.39 (0.81, 2.38) |
| Overweight or obese | **1.45 (1.05, 2.01)*** |  | **1.69 (1.02, 2.91)*** | **1.58 (1.16, 2.17)*** |
| Income < $20,000 | **3.77 (2.55, 5.62)*** | **4.56 (2.91, 7.24)*** | **5.07 (2.90, 9.09)*** | **3.82 (2.60, 5.65)*** |
| Income $20,000-$39,999 | **2.61 (1.77, 3.88)*** | **2.37 (1.480, 3.80)*** | **2.07 (1.11, 3.89)*** | **2.10 (1.44, 3.07)*** |
| Income $40,000-$59,999 | 1.36 (0.88, 2.06) | 1.24 (0.71, 2.12) | 1.09 (0.49, 2.279) | 1.122 (0.81, 1.81) |
| ^a^ Distance to roadway classified as < 300 m and > 300 m from the nearest primary or secondary road.  ^b^ Results reported are for adjusted models, which include sex, race, smoking status, body mass index, and income category as covariates  ^c^ Reference group is neither responder, > 300 m from road, Caucasian, female, nonsmoker, normal or underweight body mass index, and income > $60,000  ^d^ OR reported in this row indicate the odds of a member of the reference group having an asthma exacerbation.  *Indicates significance at *p* < 0.05  ^+^ Indicates *p* < 0.10 | | | | |

| **Table S16. Sensitivity analysis: Asthma exacerbations, responder status, and 400m distance to road**^a,b^ | | | | |
| --- | --- | --- | --- | --- |
|  | Activity limitations  OR (95% CI) | Sleeplessness  OR (95% CI) | Emergency room visits  OR (95% CI) | Any exacerbations  OR (95% CI) |
| Reference group:  Asthma diagnosis, no exacerbation^c^ | 1.00 (reference) | 1.00 (reference) | 1.00 (reference) | 1.00 (reference) |
| Reference group^d^ | **0.25 (0.17, 0.36)*** | **0.08 (0.05, 0.14)*** | **0.39 (0.020, 0.073)*** | **0.33 (0.23, 0.48)*** |
| < 400 m distance | *0.71 (0.46, 1.09)* | 0.77 (0.45, 1.27) | 0.76 (0.38, 1.30) | **0.66 (0.43, 1.01)*** |
| Hyper responder | 1.29 (0.86, 1.91) | 0.91 (0.55, 1.48) | 0.84 (0.44, 1.52) | 1.22 (0.81, 1.81) |
| Hypo responder | **0.46 (0.21, 0.96)^*^** | **0.36 (0.12, 0.91)*** | 0.47 (0.11, 1.40) | **0.50 (0.27 0.99)^*^** |
| Smoker | *1.26 (0.95, 1.69)^+^* | **1.72 (1.22, 2.42)*** | 1.35 (0.89, 2.06) | 1.22 (0.93, 1.62) |
| Male | **0.64 (0.45, 0.89)*** | **0.38 (0.24, 0.59)*** | 0.77 (0.46, 1.26) | **0.56 (0.40, 0.78)*** |
| African American | 0.76 (0.53, 1.07) | **1.56 (1.06, 2.27)*** | **1.66 (1.05, 2.61)*** | 0.95 (0.67, 1.33) |
| Other races | 1.19 (0.68, 2.07) | 1.13 (0.55, 2.18) | 0.92 (0.33, 2.15) | 1.41 (0.82, 2.43) |
| Overweight or obese | **1.44 (1.04, 2.00)*** | **1.75 (1.17, 2.66)*** | **1.69 (1.02, 2.91)*** | **1.57 (1.15, 2.16)*** |
| Income < $20,000 | **3.81 (2.57, 5.68)*** | **4.59 (2.92, 7.29)*** | **5.08 (2.90, 9.11)*** | **3.87 (2.63, 5.73)*** |
| Income $20,000-$39,999 | **2.63 (1.78, 3.90)*** | **2.37 (1.48, 3.81)*** | **2.06 (1.11, 3.87)*** | **2.11 (1.44, 3.09)*** |
| Income $40,000-$59,999 | 1.36 (0.89, 2.08) | 1.24 (0.71, 2.65) | 1.10 (0.50, 2.29) | 1.123 (0.82, 1.83) |
| ^a^ Distance to roadway classified as < 400 m and > 400 m from the nearest primary or secondary road.  ^b^ Results reported are for adjusted models, which include sex, race, smoking status, body mass index, and income category as covariates  ^c^ Reference group is neither responder, > 400 m from road, Caucasian, female, nonsmoker, normal or underweight body mass index, and income > $60,000  ^d^ OR reported in this row indicate the odds of a member of the reference group having an asthma exacerbation.  *Indicates significance at *p* < 0.05  ^+^ Indicates *p* < 0.10 | | | | |

| **Table S17. Sensitivity analysis: Asthma exacerbations, responder status, and 500m distance to road**^a,b^ | | | | |
| --- | --- | --- | --- | --- |
|  | Activity limitations  OR (95% CI) | Sleeplessness  OR (95% CI) | Emergency room visits  OR (95% CI) | Any exacerbations  OR (95% CI) |
| Reference group:  Asthma diagnosis, no exacerbation^c^ | 1.00 (reference) | 1.00 (reference) | 1.00 (reference) | 1.00 (reference) |
| Reference group^d^ | **0.25 (0.17, 0.36)*** | **0.09 (0.05, 0.14)*** | **0.041 (0.0208, 0.08)** | **0.33 (0.23, 0.48)** |
| < 500 m distance | 0.84 (0.57, 1.23) | 0.81 (0.51, 1.28) | *0.62 (0.32, 1.11)^+^* | 0.75 (0.51, 1.09) |
| Hyper responder | 1.28 (0.86, 1.91) | 0.92 (0.55, 1.48) | 0.85 (0.45, 1.53) | 1.22 (0.82, 1.81) |
| Hypo responder | **0.46 (0.20, 0.95)^*^** | **0.36 (0.12, 0.90)*** | 0.46 (0.11, 1.39) | **0.49 (0.23, 0.98)^*^** |
| Smoker | *1.27 (0.95, 1.69)^+^* | **1.72 (1.23, 2.42)*** | 1.235 (0.88, 2.06) | 1.22 (0.92, 1.62) |
| Male | **0.64 (0.45, 0.88)*** | **0.36 (0.24, 0.59)*** | 0.77 (0.46, 1.26) | **0.56 (0.41, 0.78)*** |
| African American | 0.76 (0.53, 1.06) | **1.72 (1.06, 2.27)*** | **1.66 (1.20, 2.89)*** | 0.94 (0.67, 1.32) |
| Other races | 1.19 (0.68, 2.06) | 1.14 (0.55, 2.20) | 0.94 (0.34, 2.19) | 1.42 (0.82, 2.43) |
| Overweight obese | **1.45 (1.05, 2.01)*** | **1.75 (1.18, 2.66)*** | **1.69 (1.02, 2.91)*** | **1.58 (1.15, 2.17)*** |
| Income < $20,000 | **3.76 (2.54, 5.60)*** | **4.57 (2.91, 7.24)*** | **5.10 (2.91, 9.15)*** | **3.81 (2.59, 5.64)*** |
| Income $20,000-$39,999 | **2.60 (1.76, 3.86)*** | **2.36 (1.48, 3.79)*** | **2.07 (1.11, 3.89)*** | **2.09 (1.43, 3.06)*** |
| Income $40,000-$59,999 | 1.36 (0.89, 2.07) | 1.24 (0.71, 2.13) | 1.08 (0.49, 2.27) | 1.22 (0.81, 1.82) |
| ^a^ Distance to roadway classified as < 500 m and > 500 m from the nearest primary or secondary road.  ^b^ Results reported are for adjusted models, which include sex, race, smoking status, body mass index, and income category as covariates  ^c^ Reference group is neither responder, > 500 m from road, Caucasian, female, nonsmoker, normal or underweight body mass index, and income > $60,000  ^d^ OR reported in this row indicate the odds of a member of the reference group having an asthma exacerbation.  *Indicates significance at *p* < 0.05  ^+^ Indicates *p* < 0.10 | | | | |
